# Late-onset sarcoidosis in a patient with gastric mucosa-associated lymphoid tissue non-Hodgkin lymphoma: A case report

**DOI:** 10.3892/ol.2014.2241

**Published:** 2014-06-12

**Authors:** MARTINA TORCHIO, GIORGIO BOTTARO, GIAMPIERA BERTOLINO, GIUDITTA COMOLLI, BARBARA DAL BELLO, ROSANGELA INVERNIZZI, MARCO DANOVA

**Affiliations:** 1 Department of Internal Medicine and Medical Oncology, Civic Hospital of Vigevano, Pavia I-27029, University of Pavia, Pavia I-27100, Italy; 2 Department of Internal Medicine, University of Pavia, Pavia I-27100, Italy; 3 Department of Biotechnology Research Laboratory and Microbiology and Virology, IRCCS Foundation Policlinico San Matteo, University of Pavia, Pavia I-27100, Italy; 4 Department of Pathology, University of Pavia, Pavia I-27100, Italy

**Keywords:** mucosa-associated lymphoid tissue, sarcoidosis, sarcoidosis-lymphoma syndrome, granulomatous disorders, malignancy

## Abstract

The simultaneous presence of hematological malignancies and sarcoidosis, defined as sarcoidosis-lymphoma syndrome, has been reported in 79 patients in the literature to date. The majority of these patients were affected by sarcoidosis and developed non-Hodgkin lymphoma or acute leukemia after 1–2 years; however, in <20 cases the malignancy developed first. This report presents the case of an 83-year-old male with a clinical history of *Helicobacter pylori*-positive gastric mucosa-associated lymphoid tissue lymphoma. The patient developed sarcoidosis 10 years after the first diagnosis, which caused the diagnostic work-up and differential diagnosis between a lymphoma relapse and *de novo* sarcoidosis to be challenging.

## Introduction

The correlation between malignancy and sarcoidosis remains controversial (1). To the best of our knowledge, the first attempt to quantify the incidence of this phenomenon occurred in a retrospective study, which included 2,544 subjects presenting with sarcoidosis (2). Additional evidence that was obtained from retrospective clinical studies (3,4) and genetic linkage-analyses (5,6) focused on the association of sarcoidosis and malignancies and indicated a potential etiological correlation between the two clinical entities. These data have enabled, in certain cases, the diagnosis of a rare pathological condition defined as sarcoidosis-lymphoma syndrome (SLS) (1). Typically, SLS refers to patients with chronic, active sarcoidosis onset at a median age that is greater than that of the non-oncologic population, who develop a lymphoma [most frequently Hodgkin’s lymphoma (HL)] after 1–2 years. A small number of patients with SLS and a clinical history of lymphoma or leukemia present with late-onset sarcoidosis (7,8,9).

In conventional SLS, the sarcoidotic pathway with the subsequent chronic inflammatory immune status, may determine a dysregulation of the cell immunological pattern, permitting the onset of a lymphomatous/leukemic disorder. By contrast, the physiopathology of the non-conventional presentation of SLS remains unclear (10).

This report presents a case of sarcoidosis with prevalent hepatic and cutaneous localizations, which developed 10 years after the diagnosis of *Helicobacter pylori* (*H. pylori*)-positive gastric mucosa-associated lymphoid tissue (MALT) lymphoma. The patient was treated with *H. pylori*-eradicating therapy only.

## Case report

This report presents an 83-year-old male, who in 2001, in the absence of any significant pre-existing pathology and presenting with gastritis-like symptoms, was diagnosed with *H. pylori*-positive gastric MALT lymphoma. The complete disease staging was negative due to the pathological presence of lymphadenomegaly in the neck, chest, abdomen and pelvis, as well as secondary involvment in the liver, spleen and bone marrow. The patient was treated only with a specific *H. pylori*-eradicating therapy, which resulted in a complete clinical remission with the eradication of the bacteria and histologically documented normal gastric mucosa. Written informed consent was obtained from the son of the patient for publication of this case report.

**Figure 1:**
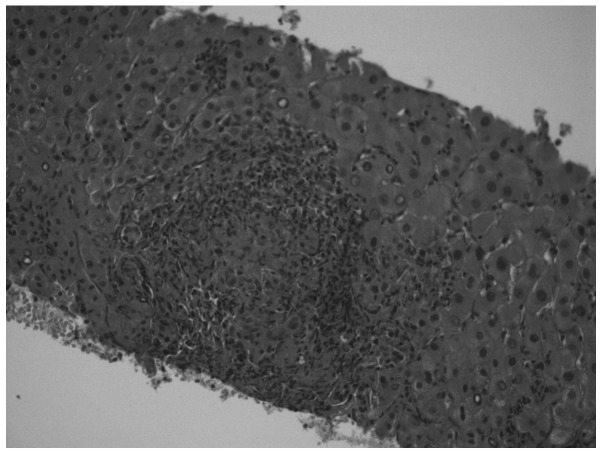
Liver biopsy containing a well-circumscribed epithelioid granuloma (hematoxylin and eosin stain; magnification, ×200).

**Figure 2:**
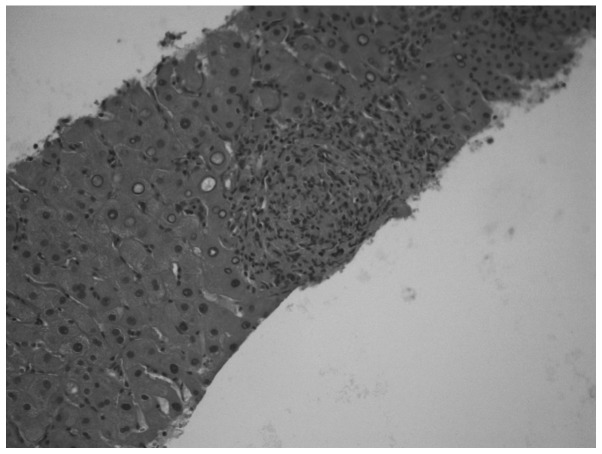
Liver biopsy section demonstrating a discrete, non-necrotizing epithelioid granuloma (hematoxylin and eosin stain; magnification, ×400).

The patient underwent regular clinical and instrumental follow-up examinations that revealed no disease relapse. In December 2010, the patient presented with a recent onset of asthenia, nausea, dyspepsia and moderate weight loss. Based on the suspected disease relapse, an endoscopic examination was performed, which was negative for non-specific lesions and lymphoid infiltration, and revealed a mild-grade chronic gastritis pattern. An ultrasound liver examination identified multiple solid heterogeneous lesions that were confirmed by computed tomography (CT) scanning, which also revealed multiple abnormal mediastinal and retroperitoneal lymph nodes. Routine blood tests, including tumor marker assays, lactate dehydrogenase and β2 microglobulin were considered to be in the normal range. The circulating leukocyte pattern showed a non-significant increment in the CD8^+^ cell subset, diffuse presence of activated CD3^+^/HLA-DR T-cells and rare B-cells. Positron emission tomography was negative for ipercaptant lesions/lymph nodes and for suspected lymphomatous localizations. A bone biopsy excluded lymphoma relapse in the bone marrow. An ultrasound-guided biopsy was performed on one of the major hepatic nodules and non-specific granulomatous epithelial-like hepatitis was diagnosed (Figs. 1 and 2). Staining for acid-fast bacilli and fungi was performed and excluded these organisms as causative agents. Autoimmunity tests, the viral hepatitis screening panel, virus and bacteria-associated infection tests, and the Mantoux test were all negative.

The clinical condition of the patient as well as the routine blood analyses remained stable until the end of April 2011. The patient then presented with a mild-grade fever, and rapid and significant weight loss associated with nodular lesions (with an erythema nodosum-like pattern) and a pruritus-causing cutaneous-rash predominantly localized to the legs. The test results for bacterial or viral infections were consistently negative. Hepatic lesions and lymph node characteristics were stable following the CT examination, however, blood analyses revealed that the angiotensin-converting enzyme (ACE) serum level was higher (156 U/l) compared with that of the normal level (8–52 U/l).

The patient was admitted to the Department of Internal Medicine at the Civic Hospital Vigevano (Pavia, Italy) at the beginning of June. Blood analyses revealed mild anemia (a haemogloblin concentration of <9.0 g/dl) with negative fecal hemoccult test findings, high β2 microglobulin values (11.2 mg/l) and the ACE serum level was 235 U/ml. The upper gastrointestinal endoscopy was negative for ulcerative lesions and gastric bleeding, and only showed a chronic erythematous gastritis pattern. The CT findings were comparable to the results of CT scans performed during the previous six months as follows: Liver lesions appeared stable, lung parenchyma remained negative for disease secondary localizations and the abnormal lymph nodes were unchanged. The results of the skin biopsy indicated granulomatous non-caseating lesions with a psoriasis-like pattern.

Oral corticosteroid therapy (37.5 mg prednisone per day) was administered and after one week a progressive reduction of symptoms was noted. Complete resolution of the skin lesions and the disappearance of the fever were subsequently achieved. The patient was discharged from hospital, however, continued with the steroid therapy for two weeks, which was followed by a progressive dosage reduction. Immediately following the first tapering of prednisone (25 mg prednisone per day), the fever reappeared with the same characteristics. All of the blood tests, including the repeated searches for bacterial or viral agents, were negative and the CT scan was stable. The results from fresh hepatic and bone marrow biopsies were negative for lymphomatous lesions and cell infiltraion. The fever disappeared when the previous steroid dosage was resumed.

The clinical conditions of the patient remained stable until the beginning of September 2011, when, despite steroid therapy, they rapidly worsened with a reappearance of a fever and the multiple organ dysfunction/failure syndrome, which resulted in the patient succumbing to acute renal failure in November 2011.

## Discussion

Bichel and Brincker (1,11–13) were the first to examine cases of sarcoidosis that were co-presenting with malignant tumors identified in the Danish Cancer and Sarcoidosis Registries (14). Bichel and Brincker noted a higher incidence of lymphoma in the sarcoidosis population compared with that in the general population. Thus, SLS was used to describe a pathological entity that was characterized by the onset of a lymphoma (most commonly HL) following a previous diagnosis of sarcoidosis, whose development was noted ~10 years later, in comparison to a diagnosis of sarcoidosis that was made in the general population.

Two previous studies failed to confirm the association between sarcoidosis and lymphoma (15,16). Furthermore, in an attempt to quantify the association between non-HLs (NHLs) and various autoimmune and chronic inflammatory disorders, Mellemkjaer *et al* (17) conducted an analysis on >25,000 patients with sarcoidosis that were obtained from the Swedish and Danish Cancer Registries (14). A notable increase in the risk of NHL was observed in patients with a previous history of sarcoidosis (odds ratio, 1.9). A previous linkage analysis supports this association and indicated that ≥25% of patients with sarcoidosis may develop a malignancy (5,6).

Based on the abovementioned observations, certain investigators aimed to clarify, from a pathogenetic perspective, the significant association between sarcoidosis and malignancies, and proposed an immunopathogenetic model. Noor and Knox (10) observed that sarcoidosis is invariably accompanied by significant alterations in the immune system, predominantly hyperstimulation and the increased mitogenesis of B and T lymphocytes. This may predispose the subject to the development of lymphoid malignancies.

In a small percentage of SLS cases the diagnosis of lymphoma was given prior to the onset of sarcoidosis. In these rare cases, it was indicated that anticancer chemotherapy contributed to the worsening of the clinical manifestations of the underlying sarcoidosis (2).

In this context, the patient in the current study presented certain noteworthy characteristics as follows: i) The unusual presentation of NHL, which preceded sarcoidosis and was not treated with chemotherapy; ii) the long interval between the two diagnoses; iii) the particular type of NHL, a gastric MALT lymphoma that, to the best of our knowledge, was previously reported in <10 patients (2,18,19); and iv) the unusual presentation of sarcoidotic lesions, in particular, the hepatic and cutaneous manifestations (a psoriasis-like sarcoidosis pattern). These findings were not classically pathognomonic of sarcoidosis and complicated the differential diagnosis between other possible causes of granulomatous disorders (20,21). Skin manifestations in sarcoidosis occur in ~20–35% of patients and are typically present at the onset of sarcoidosis (22).

In conclusion, due to the long interval that elapsed between the two pathological onsets and the abovementioned unusual localizations, the differential diagnosis between a lymphoma relapse and a *de novo* sarcoidosis was challenging.
